# Use of Technologies for the Acquisition and Processing Strategies for Motion Data Analysis

**DOI:** 10.3390/biomimetics10050339

**Published:** 2025-05-20

**Authors:** Andres Emilio Hurtado-Perez, Manuel Toledano-Ayala, Irving A. Cruz-Albarran, Alejandra Lopez-Zúñiga, Jesús Adrián Moreno-Perez, Alejandra Álvarez-López, Juvenal Rodriguez-Resendiz, Carlos A. Perez-Ramirez

**Affiliations:** 1 Division de Estudios de Posgrado, Facultad de Ingeniería, Universidad Autónoma de Querétaro, Cerro de las Campanas S/N, Querétaro 76010, Mexico; ahurtado19@alumnos.uaq.mx (A.E.H.-P.); toledano@uaq.mx (M.T.-A.); adrian.moreno@uaq.mx (J.A.M.-P.); alejandra.alvarez@uaq.mx (A.Á.-L.); 2 Tequexquite, Centro de Investigación y Desarrollo Tecnológico para la Accesibilidad e Innovación Social, Facultad de Ingeniería, Universidad Autónoma de Querétaro, Campus Aeropuerto, Carretera a Chichimequillas S/N, Ejido Bolaños, Querétaro 76140, Mexico; alopez145@alumnos.uaq.mx; 3 C.A. Sistemas de Inteligencia Artificial Aplicados a Modelos Biomédicos y Mecánicos, Facultad de Ingeniería, Universidad Autónoma de Querétaro, Campus San Juan del Rio, Rio Moctezuma 249, Col. San Cayetano, San Juan del Río 76807, Mexico; irving.cruz@uaq.mx; 4 C.A. Sistemas de Inteligencia Artificial Aplicados a Modelos Biomédicos y Mecánicos, Facultad de Ingeniería, Universidad Autónoma de Querétaro, Campus Aeropuerto, Carretera a Chichimequillas S/N, Ejido Bolaños, Querétaro 76140, Mexico

**Keywords:** healthcare innovation, healthcare data analysis, biomechanical analysis, inertial measurement unit (IMU), motion-capture system (MCS)

## Abstract

This review provides an in-depth examination of the technologies and methods used for the acquisition and processing of kinetic and kinematic variables in human motion analysis. This review analyzes the capabilities and limitations of motion-capture cameras (MCCs), inertial measurement units (IMUs), force platforms, and other prototype technologies. The role of advanced processing techniques, including filtering and transformation methods, and the increasing integration of artificial intelligence (AI) and machine learning (ML) for data classification is also discussed. These advancements enhance the precision and efficiency of biomechanical analyses, paving the way for more accurate assessments of human movement patterns. The review concludes by providing guidelines for the effective application of these technologies in both clinical and research settings, emphasizing the need for comprehensive validation to ensure reliability. This comprehensive overview serves as a valuable resource for researchers and professionals in the field of biomechanics, guiding the selection and application of appropriate technologies and methodologies for human movement analysis.

## 1. Introduction

Broadly speaking, biomechanics is defined as the field that studies the mechanical aspects of living organisms, i.e., the human body [1]. In particular, biomechanics-based analysis has found applications in the following [2,3]:

1. Laboratory-based research environments: As expected, these scenarios have controlled conditions, as they are used to assess the accuracy and precision, among other properties, of either measurement instruments or methodologies. For instance, gait studies require this type of environment for performing their analysis [2,3,4].
2. Clinical and rehabilitation environments: A patient-centered scenario is considered, where comfort, safety, and real-time feedback are the interest parameters that need to be estimated once the rehabilitation procedure is employed. In this sense, biomechanical-based technologies are often selected by considering their ease of use, non-invasiveness, and ability to track recovery progress [5,6], allowing the assessment of the therapy performance without requiring the patient opinion regarding its own progress.
3. Sport-based environments: These applications require portability, robustness, and minimal setup time. Wearables and wireless devices are commonly used to monitor during training, competition, or test [7,8].
4. Day-to-day environments: Activities such as activity monitoring, ergonomics, and preventive healthcare are of particular interest. Hence, the technologies employed in these contexts need to be compact and user-friendly, as they are designed for long-term use without expert supervision [9].

**Figure 1:**
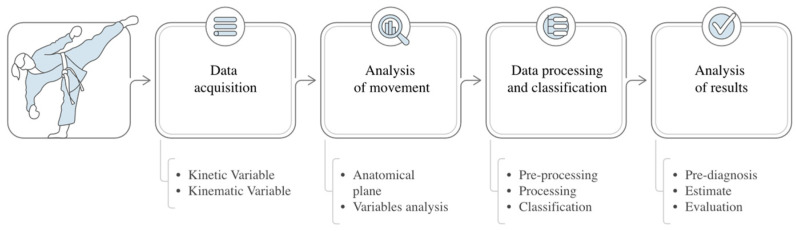
Employed methodology for motion analysis. The arrows indicate the flow sequence to execute the proposed steps.

Figure 1 shows a general diagram for a methodology that performs a biomechanical analysis.

From the figure, it can be seen that four steps are employed. The first one, data acquisition, uses technology to measure the movement and/or the associated variables. Next, the movement-analysis step performs the data interpretation; in other words, data are no longer numbers and are seen as a motion analysis. After that, data processing and classification is usually performed in three stages: preprocessing, processing, and classification. Preprocessing stage performs the data cleaning and normalization for its proper use and analysis; the processing data stage estimates the required parameters that are indicated by the performed movement analysis. If a movement detection is required, the classification stage assigns the class to which the measured data belongs. To this purpose, algorithms based on artificial intelligence (IA), statistical, thresholds, or specific criteria are the employed ones.

It should be noted that to measure different variables related to human movement to perform the abovementioned tasks, several technologies have been employed, where motion-capture systems (MCSs), inertial measurement units (IMUs), and force platforms are the most employed ones, as they allow us to capture the most information regarding the execution of the movements or trajectories [10]. In this sense, some comments can be made. MCCs are considered the gold standard in the measurement of motion analysis [10,11]. These technologies employ cameras to obtain motion-analysis data frame to frame, using images acquired from recorded video. IMU-based sensors use gravitational properties as measurement strategies; for this purpose, accelerometers and gyroscopes are the sensors that acquire data through the movement of a small mass that is suspended and referenced to the gravitational acceleration [12,13]. On the other hand, force platforms use the measurement of the impact or pressure applied to their surface by employing the induced deformation or the impact force, among other strategies [14,15].

Once the data are acquired, the information that can be extracted is usually denoted as kinetic or kinematic variables. Kinetic variables are used in dynamic motion analysis to understand the causes that generate the movement, where some examples are the measurement of force, energy, power, and work [16], among others. On the other hand, kinematic variables measure the body movement, where displacement, velocity, acceleration, position, and time are the measured variables [9].

To obtain the information required to elaborate the claims in this paper, exhaustive research was performed using the scholar databases Scopus and Google Scholar, where papers from 2018 to 2025 are the selected ones, as the idea of this review was to obtain a unique paper that contains the following:

- A practical strategy to find the technologies employed to acquire motion analysis variables in different activities for performing biomechanical analysis.
- An effective method to identify the different techniques for the preprocessing, processing, and classification of motion-analysis data.
- A new strategy to classify validation results in motion analysis.
- Recommendations and strategies for the use of technologies and techniques for preprocessing, processing, and classification in motion analysis.

**Figure 2:**
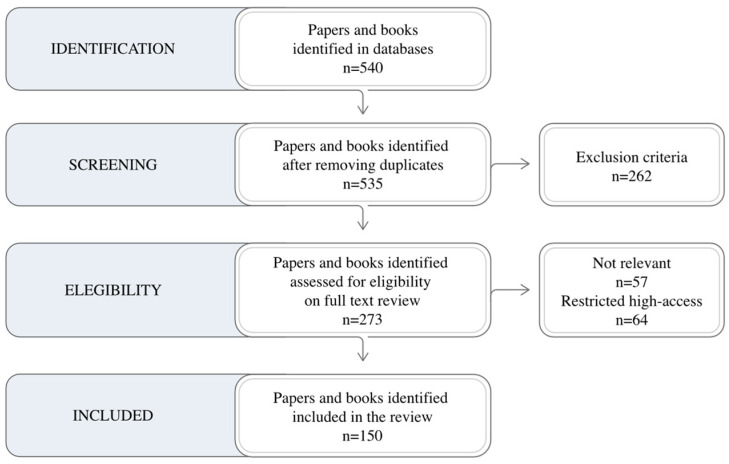
Employed PRISMA flow diagram for this review.

Bearing this in mind, Figure 2 shows the PRISMA flow diagram employed in this review. This diagram is the reference methodology due to the capability to discriminate papers, giving a structured way to select the appropriate ones. Following the diagram the inclusion criteria are English-written papers and Q1 or Q2 journals.

**Figure 3:**
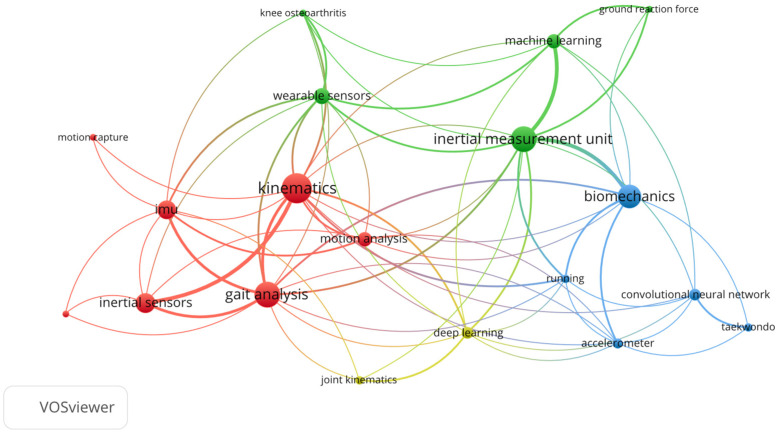
Bibliometric network of papers employed in this review.

Using the abovementioned PRISMA methodology, 151 papers and books fulfilled the criteria. After that, a Bibliometric network using the papers’ keywords were obtained to determine the most common terms. To this purpose, the software VOSviewer 1.6.20 was used, as it generates a visual representation of the how the papers’ aims are related using their keywords. In this sense, it can be observed in Figure 3 that the most concurrent keywords meet the objectives denoted for this review.

To the best of the authors’ knowledge, different reviews have been published over the years [9,10,11,16], yet a holistic overview that considers all the aspects of the motion analysis, i.e., from the signal acquisition to its processing, is still missing. This paper presents a State-of-the-Art review of biomechanical analysis, focusing on the technologies employed for motion-data acquisition and how they are deployed in different activities, such as clinical rehabilitation, sports performance, and everyday monitoring.

This review explores the data-processing and -classification strategies, highlighting trends in the use of artificial-intelligence (AI), machine-learning (ML), and deep-learning (DL) methods. This perspective highlights alternative approaches in motion analysis research, where the emphasis is placed on the robustness of the methodology and the measurement accuracy; further, guidelines regarding the technology usage, as well as the advantages and disadvantages for both the signal-processing and classification stages are also presented. The goal of this paper is to present a gentle yet complete introduction to the development of biomechanical-based applications.

## 2. Technologies Employed to Acquire Motion-Based Variables

This section presents the main technologies used in motion analysis, and their respective advantages and disadvantages are presented and discussed. This section also describes the applications and physical scenarios in which they are deployed.

### 2.1. Motion-Capture Systems (MCSs)

Motion-capture systems (MCSs) are high-precision optical-based systems that employ calibrated cameras, which are deployed in a defined spatial configuration to accurately reconstruct human motion in the three-dimensional space. These systems use the triangulation strategy to reconstruct or calculate either the trajectories or the variables of interest. Each camera captures two-dimensional projections of reflective markers placed in specific corporal positions [10,16,17,18]; therefore, this setup allows the temporal analysis of movement patterns frame by frame, making MCSs ideal for applications requiring both high spatial and temporal resolution, such as gait analysis, sport performance evaluation, and clinical diagnostics [2,3,4,19,20,21,22,23,24,25,26,27,28,29].

It should be noted that a wide range of optical MCSs are identified, reflecting the diversity and evolution of available technologies in both research and clinical applications. In this sense, systems such as Vicon (e.g., Vicon T40, Vero, Vantage V5, Nexus V2, and MX-F40), Qualisys (Oqus 7+, Oqus 4-series, and QualisysTrack Manager), and Motion Analysis Corp (e.g., Hawk, Raptor-4, and Smart-Dx) are frequently employed. These systems are often employed in laboratory environments, since a robust calibration, multi-camera configurations, and compatibility with biomechanical modeling software allow for detailed 3D motion reconstruction, and quantitative biomechanical analyses are often required.

Additionally, other complementary or hybrid setups are noted, including low-cost camera systems (e.g., GoPro Hero 3, and regular webcams), depth sensors (e.g., Microsoft Kinect V1/V2, Azure Kinect, and Orbbec Astra), and marker-less tracking platforms integrated with photogrammetry or stereoscopic vision systems (e.g., BTS Bioengineering and Dalsa Falcon). They are particularly relevant in for practical environments, where mobility, setup simplicity, and cost-effectiveness are prioritized over sub-millimetric spatial accuracy.

**Figure 4:**
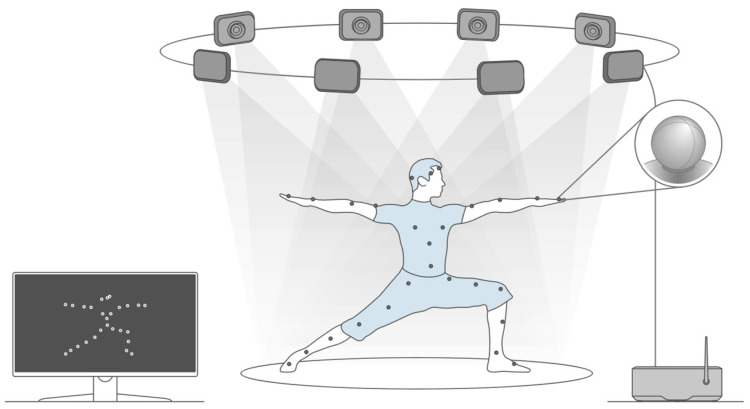
Marker-based motion capture-system setup.

Figure 4 illustrates a typical example of the use of an MCS in a controlled environment. It depicts the typical use, with retroreflective markers placed at key anatomical landmarks, which are tracked by multiple infrared cameras positioned around the capture volume. This configuration allows for the real-time acquisition of three-dimensional motion trajectories, which are subsequently processed to extract biomechanical parameters such as joint angles, segmental velocities, and inter-limb coordination patterns, among others. These setups are commonly used found in laboratories specializing in gait analysis, sport biomechanics, and rehabilitation.

To provide a clearer understanding of the diversity and applicability of the systems, Table 1 presents a qualitative analysis of the most-used MCS technologies. It should be noted that the table presents the employed sensors according to their type and recommended environment.

Gait analysis is the application that uses MCSs, due to its spatial tracking and full-body kinematic reconstruction, since they preserve the patient’s natural biomechanics during data acquisition [10,16,17,18]; further, MCSs can accurately capture the lower motion [2,3,4,16,19,20,21,22,23,24,25,26,27,28,29]. Similarly, in running studies, MCSs are frequently selected since they can handle dynamic transitions [16,26,27,30,31]. In more complex or high-speed activities, such as cutting maneuvers [32,33], where sudden directional changes are involved, MCSs enable the fine capture of instantaneous motion without restricting the fluidity. Applications of MCSs have been reported in squats [34,35], martial arts [36,37], baseball [37], football [38], and upper and lower limbs [39,40,41].

### 2.2. Inertial Measurement Units (IMUs)

Inertial Measurement Units (IMUs) are embedded sensor systems designed for estimating human movement parameters by capturing three-dimensional linear acceleration, angular velocity, and magnetic field vectors. To this purpose, triaxial accelerometers, gyroscopes, and magnetometers, are integrated into compact hardware platforms, where built microelectromechanical system (MEMS) technology is the preferred option for the sensor development, as MEMS-based sensors have a high signal-to-noise ratio (SNR), low power consumption, and reduced size [12,42], which are desirable features for portable systems.

IMUs are typically placed on body segments or near joints, such as the tibia, femur, forearm, or thorax, depending on the movement to be analyzed. In this sense, proper placement is essential for accurately tracking segmental motion, thus ensuring repeatability across trials or subjects. In general terms, the placement strategy depends on the biomechanical model adopted, which is aligned with the desired degrees of freedom (DoFs). After data acquisition, the raw signals are processed to obtain reliable estimates of segment orientation and movement. The resulting orientation, commonly expressed in quaternions or Euler angles, are integrated into kinematic models that represent the body as a chain of interconnected rigid segments. These models enable reconstruction of motion variables such joint angles, angular velocities, and intersegmental coordination [43].

**Figure 5:**
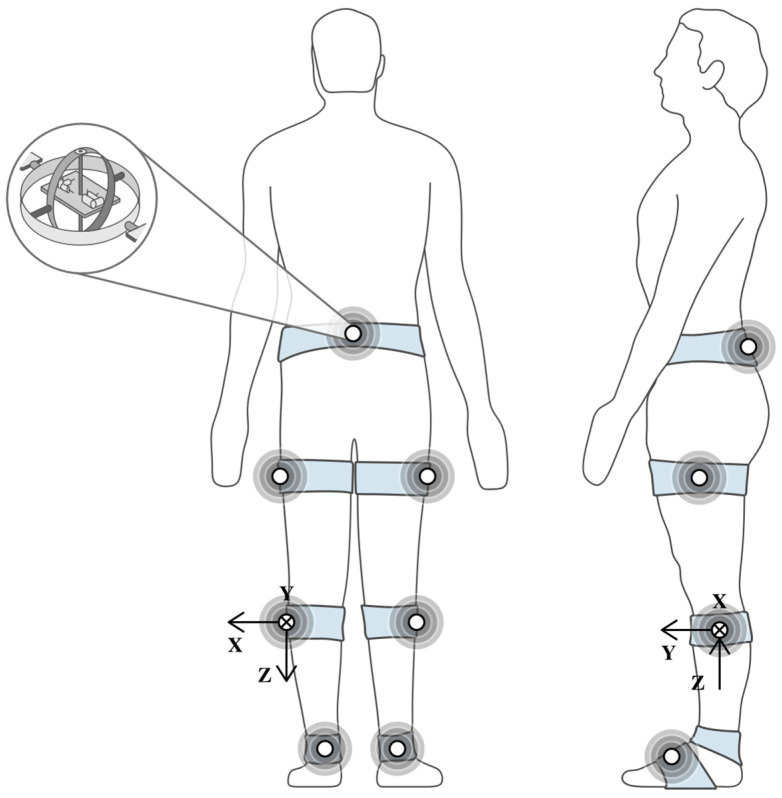
Schematic representation of an IMU-based motion analysis setup.

It is worth noticing that when IMUs are synchronized and distributed on multiple body segments, a robust and versatile option for motion analysis in clinical assessment can be achieved. IMUs’ application fields include sport science and wearable technology, enabling either real-time or offline feedback systems [20,43,44,45,46]. Figure 5 illustrates the typical implementation of an IMU-based motion-tracking system.

Each IMU captures both the linear acceleration and angular velocity relative to its local frame of reference. The zoomed-in region represents the internal components, indicating how the elements respond to the mechanical deflection caused by motion. MEMSs generate their signal using capacitive-based transductors. In this sense, this configuration enables the reconstruction of segmental orientation and joint dynamics using fusion algorithms and kinematic modeling [43].

To illustrate the diversity of commercial systems used in IMUs’ motion analysis, Table 2 presents a qualitative analysis of their main features. The systems are organized according to their type and recommended application environment. This classification includes high-end commercial platforms such as Xsens and Noraxon, which are widely used in clinical and research environments due to their robust communication and integration with a biomechanical modeling software program [14]. Further, wearables; sport-oriented systems such as GaitUp, MyoMotion, and IMeasuredU; and custom-built or research-focused IMUs, such as those developed by Portabilities GmbH or LORD MicroStrain, are also presented, demonstrating the flexibility and adaptability of IMU technology for specialized applications.

From the table, it can be seen that IMUs have gained significant traction in motion analysis due to their affordability and ability to operate without the need for complex infrastructure, making them attractive for performing gait analysis, where multiple studies have reported their effective use for capturing data [2,3,4,12,17,19,20,22,23,25,28,29,44,45,46,47,48,49,50,51,52,53,54,55,56]. IMUs have also proven effective in activities requiring precise temporal and spatial resolution, such as cutting maneuvers [18,32,33] and upper limb-motion analysis [40,57,58,59,60,61,62,63]. In addition, IMUs have been employed in a wide range of dynamic activities, including martial arts [36,64,65,66], running [13,67,68,69,70], football [71], lower-limb motion [61,63,72,73,74], and swimming [11,43]. IMUs have also been applied to neck-motion studies [5,6,75], where small sensor sizes and non-invasive configurations are essential both for the patient’s comfort and safety.

### 2.3. Force Platforms

**Figure 6:**
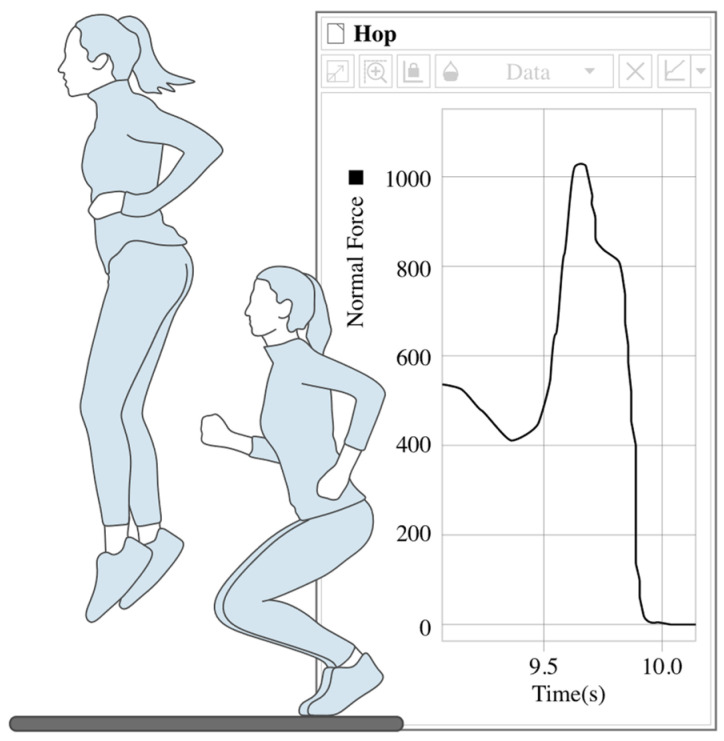
Force platform utilization.

Force platforms are electromechanical transducers designed to measure the ground reaction forces (GRFs) generated when a body applies force to the surface. These devices usually employ strain gauge-based or piezoelectric-based transducers embedded in rigid plates, making them capable of detecting forces and moments along three orthogonal axes. The operating principles relies on Newtons’ third law, where the platform captures the reactive force exerted by the ground when a subject exerts pressure through contact (typically via the feet) [2,3,4,16,34]. Figure 6 illustrates the practical implementation of force platforms in biomechanical-analysis scenario.

The subject is instructed to perform a specific motion (e.g., gait, jumping, or squatting) on the platform surface, which registers the ground reaction forces generated upon contact. These forces are recorded and can be analyzed independently or along with kinematic data obtained from IMUs or MCS.

Table 3 provides a comparative overview of the commercial systems. Kistler platforms models are often the most used since their piezoelectric sensors can capture multi-axis ground reactions forces at high sampling rates [2,3,29]. The robustness and validated performance made them ideal for gait and impact relation assessment analysis in clinical and research environments. Other systems include those from Bertec and AMTI, which are strain gauge-based technology. These platforms offer high sensitivity and support for measuring forces and torques in three dimensions, making them suitable for postural control, sport science, and balance studies. BTS platforms integrate with optoelectronic systems from BTS bioengineering, enabling synchronized acquisition of force and motion data.

On the other hand, force platforms are devices that are usually employed as a reference standard when using MCS or IMUs for measuring forces during human movements [2,3,4,16,34]. Their high precision and ability to capture GRFs in three dimensions make them essential tools in biomechanical analysis, particularly for validating or complementing data from kinematic systems [29,34,75].

They have been applied in gait analysis [2,3,29], where temporal forces profiles provide insights into walking dynamics, load distribution, and asymmetries. These metrics are important in clinical assessments to identify pathological gait patterns or to evaluate progress in rehabilitation programs. In addition, force platforms are also commonly used in squat analysis [34], where they enable the analysis or vertical GRFs, balanced control, and interlimb coordination, among others [29,34,35].

### 2.4. Other Technologies

Although MCSs, IMUs, and force platforms are the most employed technologies for motion acquisition in biomechanical analysis [2,3,4,16,34], recently, researchers have been explored alternatives, where flex sensors are a class of resistive transducers whose resistance varies as function of mechanical deformation (bending or elongation). As the sensor bends or stretches, its internal conductive path is altered, producing a noticeable change in resistance, which is proportional to the deformation [18,24,25,35,56,76]. By employing a resistance-based instrumentation circuit, such as the Wheatstone bridge or voltage dividers, the real-time tracking local movement can be performed. When flex sensors are placed over muscle groups or across joint regions, the deformation caused either by muscle contraction, segment rotation, or tendon stretch can be detected, depending on the employed setup. This detection is particularly valuable when studying isolated muscular responses, fine motor control or partial joint activity [18].

**Figure 7:**
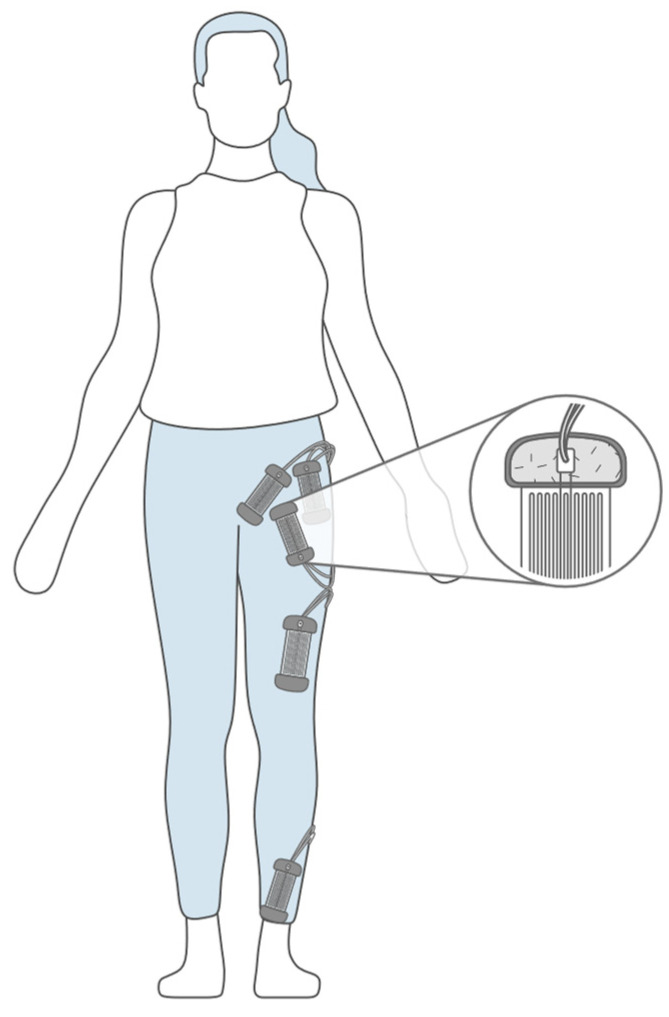
Flex sensors as an example of motion analysis technology.

Figure 7 shows a practical configuration of a flex sensor in motion analysis. The sensor is shown attached to a joint-muscle region, where the deformation produced due to cycling tension and/or compression during movement is the detected signal [18].

**Figure 8:**
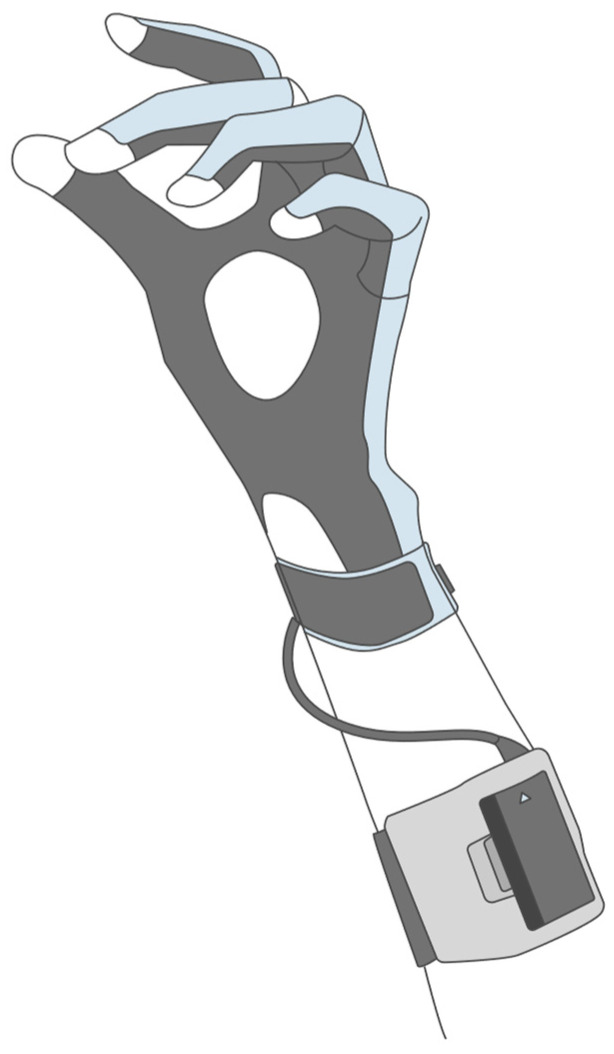
CyberGlove system^®^.

Taking advantage of the aforementioned features, CyberGlove Systems LLC (San Jose, CA, USA) developed the CyberGlove^®^ device, depicted in Figure 8. This glove incorporates multiple flex sensors embedded along the dorsal side of the fingers and hand. Each sensor is aligned with a specific joint or phalanx segment, enabling the measurement of finger flexion, abduction, and extension, allowing for the development of applications that require fine motion capture, where full-body systems like MCSs may not offer the required sensitivity or specificity for finger-level motion [18].

Along with flex sensors, other alternatives have been explored for motion analysis. In this sense, pressure-sensitive devices and its derived prototypes have been recently explored. For instance, FlexiForce o Pedar^®^ insoles have been embedded either mid or insoles to record plantar pressure distribution, step timing, and load patterns. These sensors are often arranged in arrays, providing spatial pressure maps that support applications in posture control, balance, and footwear design [24,25,45]. On the other hand, wearable stretch sensors have been recently designed to capture strain patterns and muscle dynamics, making them well-suited for analysis without restricting mobility [24,25,35,45]. Table 4 presents a summary of the abovementioned sensors.

In general terms, these technologies have been applied where traditional MCS and IMUs may be impractical. Flex sensors have been widely used in the analysis of hand and finger movements [75], offering high-resolution detection for fine motor activity. Similarly, instrumented insoles and stretch sensors have been deployed in gait analysis [24,25,45], allowing continuous monitoring of plantar pressure and gait symmetry. For squat movements [35], these systems allow the real-time tracking of the joint dynamics and load distribution without restricting the patient movement, where upper limbs [58,76] are often described. It should be noted that low-limb studies [77] benefit from the integration of pressure sensors. The adaptability and modular nature of these systems make them particularly useful in rehabilitation settings, field research, sports science, and human–machine interaction, expanding the reach of biomechanical analysis.

### 2.5. Measurement Technologies Qualitative Analysis

After the detailed examination of individual motion-capture technologies, this subsection presents a comparative synthesis to support informed decision making in system selection. Table 5 presents a comparative synthesis of the main technologies analyzed in relation to their typical application areas, advantages, and disadvantages. This summary. complements and integrates the detailed insights provided in previous Section 2.1, Section 2.2, Section 2.3 and Section 2.4, offering a practical overview to guide selection based on activity type and research constrains.

MCSs are the gold standard for high-precision biomechanical analysis in controlled laboratory environments. Their capacity to capture complex 3D movements with high spatial fidelity makes them ideal for full-body studies, including sports-based and gait studies, among others [2,3,16,19]. However, their dependency on specific and controlled environmental conditions, as well as the sensitivity for the fine-motion detection, reduces its applicability for non-controlled conditions [18,24]. In contrast, IMUs offer portability and flexibility, allowing an adequate performance under dynamic or outdoor environments [20,43,44,45]. Their integration into wearable systems enables the monitoring of segmental orientation and temporal patterns in activities such as running, martial arts, and daily life mobility (upper and lower limbs analysis). Nonetheless, their susceptibility to the drift error and calibration imposes a careful data processing [14,57,78]. On the other hand, force platforms, widely used as ground truth devices, are the preferred option for capturing ground reaction forces and torques with high accuracy; this is denoted by the reported applications in gait, impact analysis, and postural studies, particularly when synchronized with MCSs or IMUs [2,3,29,34,51]; however, they are constrained by installation requirements and their focus on surface-level force data, without tracking body kinematic.

The development of other technologies allows affirming the increasing interest in task specific, such as flex sensors, instrumented insoles, or stretch sensors. These solutions enable researchers to explore novel motion sensing paradigms, especially for fine motor tasks. Although the reported applications are noticeable, their use often implies greater development tasks without the ability to standardize the measurement strategies, which may limit their reproducibility or scalability in clinical tasks. This area should be explored to allow the development of more robust devices.

## 3. Processing, Classification, and Validation of the Measured Signals

Raw data generated from the abovementioned sensors often require signal processing to ensure accurate interpretation. In this context, this stage is commonly structured into three sequential processes: preprocessing, processing, and classification. Preprocessing refers to the cleaning normalization, and data filtering to noise removal; on the other hand, the processing stage involves either data extraction, decomposition, or transformation for calculating the desired biomechanical parameters. Depending on the study’s objective, a final classification may be implemented to categorize motion patterns, assess performance, or support clinical diagnoses. This is typically achieved using machine learning (ML) or deep learning (DL).

It should be noted that not all biomechanical analyses require classification. A vast majority only rely on statistical validation to support their results. For this reason, this review introduces a separate subsection dedicated to validation results that categorizes such studies based on the methodological approach they used to validate outcomes. This dual pathway reflects the diversity of analytical strategies in motion data analysis, underlining that both approaches are valid and relevant depending on the research objectives.

### 3.1. Preprocessing Stage

Raw data acquired from sensors such as MCS and IMUs often contain noise, artifacts, and inconsistencies due to the environmental conditions, hardware limitations, and subject variability. Preprocessing is therefore an important step that ensures the data quality of and reliability before any further processing or analysis. This stage typically includes signal filtering, normalization, and transformation to improve interpretability and comparability across datasets. It is worth nothing that some commercial technological systems include proprietary software capable of performing not only the preprocessing stage but also subsequent phases, such as data processing and classification. These integrated solutions often apply proprietary filtering algorithms, segmentation routines, and classifiers, which facilitate user interaction.

Digital Filtering

A digital filter is a system that has a frequency-selective behavior, allowing the signal processing by removing frequencies that do not have a physical reference [79]. Digital filters can be implemented using recurrent or non-recurrent approaches, with the former being the preferred choice due to the trade-off between the noise removal capability and the transition-zone size [80]. The general equation that describes the filter is denoted by the following [80]:

$$y\left[n\right]=\sum_{i=0}^{l}b_{i}x[n-i]+\sum_{i=1}^{p}a_{i}y[n-i]$$

where *l* and *p* are the coefficient numbers for the input, *x,* and output, *y*, respectively; and *a_i_* and *b_i_* are the coefficient filters, which are obtained using several approaches, with the Butterworth one being the proffered method due to its flat response [80]. It should be noted that the filter order determines transition-zone size: a higher order has a shorter transition zone between the passband and stopband; however, its magnitude must be carefully chosen since there is a risk of instability due to the algorithm computations in digital systems [80].

It should be noted that Butterworth-based implementations are the preferred option, where first-order filter is used in [81], while second-order filters appeared more frequently in studies such as [5,31,38,42,77,78]; a third-order filter was employed in [28]; fourth-order filters were used in [4,17,39,51,68,70,78,82,83,84]; a fifth-order one was used in [2,11]; an eight-order one was used in [15]; and a tenth-order one was used in [6]. Additionally, some studies applied Butterworth filters without specifying their order, as seen in [3,27,58,85].

Median filter

When the raw signal has sharp transients or non-Gaussian noise, nonlinear filtering techniques become valuable alternatives. One of the most effective among them is the median filter, which is widely used in motion-data preprocessing due to its robustness against outliers and its edge-preserving nature. The median filter operates by replacing each sample in the signal with the median value of the surrounding samples within a defined sliding window [86]. Mathematically, for a discrete time signal, $x\left[n\right]$, the output of the median filter, $y\left[n\right]$, is defined as follows [86]:

$$x\left[n\right]=median\left(x\left[n-k\right],\ldots ,x\left[n\right],\ldots ,x\left[n+k\right]\right)$$

where the filtering window is *2k + 1*. It should be noted that the median filter does not average values; instead, the central value from the sorted window is selected, effectively eliminating extreme spikes while preserving sharp changes or peaks that are common in biomechanical data motion, such as joint impact or abrupt direction changes [4,11]. In this sense, the filter has been applied to both IMUs and MCS, especially in cases where noise is irregular or highly localized [4,11].

Moving average (MA) filter

MA filter is particularly effective for eliminating high-frequency noise and revealing underlying trends in the data. This method is a linear filtering technique and operates by averaging a defined number of consecutive data points, effectively attenuating random fluctuations while preserving the overall shape of the data [87]. The moving average filter can be mathematically described as follows [88]:

$$y\left[n\right]=\frac{1}{2k+1}\sum_{i=-k}^{k}x\left[n+i\right]$$

where *k* is the window length, *x*[*n*] is the input signal, and *y*[*n*] is the filtered signal.

The low computational burden required for its implementation makes it suitable for both real-time and offline applications, where References [11,25,68,89] employ the filter to improve signal readability before subsequent processing steps, such as feature extraction.

The Stavisky–Golay (SG) filter

The SG filter is a digital filtering technique commonly employed in biomechanical data processing when it is necessary to smooth noisy data while preserving essential features such as local maxima, minima, and sharp transitions [90]. The SG filter achieves smoothing by performing local polynomial regression on a defined window of data points. Mathematically, the SG filter fits a polynomial of degree *d* to a set of 2*k* + 1 data points within a moving window centered at each time step, n, using the least-square method. The filtered output, *y*[*n*], is then the value of the fitted polynomial at the center of the window [90,91]:

$$y\left[n\right]=\sum_{i=-k}^{k}c_{i}x\left[n+i\right]$$

where *x*[*n*] is the input signal, *c_i_* represents the convolution coefficients derived from the polynomial least-square, and 2k+1 is the window size.

This method is especially useful in biomechanical context where the preservation of curvature and waveform data is critical, such as during gait and joint-angle motion or estimation from position limbs. In this context, SG filters have been implemented to optimize the balance between smoothing and features preservation [24,35,72,89,92]. It should be pointed out that a small window may fail to eliminate noise, while a large window or overly complex polynomial can introduce artifacts or over-smoothing [35,89].

Complementary filter

This filter considers the complementary characteristics of different sensors, typically accelerometers and gyroscopes, to provide a more accurate and stable estimate of system’s orientation. Gyroscopes offer high responsiveness and short-term accuracy, they are prone to drift over time; on the other hand, accelerometers provide a stable long-term reference but are sensitive to noise and dynamic acceleration. The complementary filter fuses these signals by applying a high-pass filter to the gyroscope data and a low-pass filter to the accelerometer, combining both to obtain a correct orientation estimate [93,94]:

$$\theta_{est}\left(t\right)=\alpha \left(\theta_{est}\left(t-1\right)+\omega_{gyro}\left(t\right)\cdot \Delta t\right)+\left(1+\alpha \right){\cdot \theta }_{acc}\left(t\right)$$

where $\theta_{est}\left(t\right)$ is the estimated angle at time t, $\omega_{gyro}\left(t\right)$ is the angular rate from the gyroscope, $\theta_{acc}\left(t\right)$ is the angle estimated from the accelerometer, and $\alpha \in \left[0,1\right]$ is the bending factor.

In motion analysis, complementary filters have been effectively used to estimate joint orientations or limbs trajectories where absolute orientation data are required, such as in wearable IMU systems for gait or posture assessment [73,95]. Their low computational cost and real time capability make them particularly suitable for embedded or portable systems. However, precise tuning of parameter α is essential to balance responsiveness stability.

Zero-lag filter

In applications where preserving the temporal alignment of signal features is critical, such as foot strike detection or synchronization biomechanical data, a zero-lag low-pass filter (also known as zero-phase filter) is used [96,97]. Traditional filtering schemes introduce phase delay; on the contrary, zero-lag filters apply the filtering operation forward and backward over the data, effectively cancelling out any phase shift introduced during the process. By filtering the signal in both directions, the phase delay introduces in the forward pass is exactly canceled by the reverse pass, resulting in a zero-phase (zero-lag) response [97]. In biomechanical research, this method is frequently used in conjunction with Butterworth filters to preprocess ground reaction-force data, marker trajectories, and acceleration signals, particularly when event timing is critical for analysis [8,15,18,19,30,45].

The Gaussian filter

The Gaussian filter has the ability to reduce high-frequency noise while preserving the signal overall shape. Opposite to uniform or polynomial-based filters, Gaussian filter assigns weights to data points according to Gaussian (normal) distribution, giving more importance to values near the filtering window center and gradually decreasing the influence of those further away [98,99]; further, it provides a good relationship between time-domain localization and frequency-domain attenuation, making it particularly effective for continuous motion signals such as joint angle trajectories or center of mass displacement [100]. This results in smooth transitions and minimal signal distortion [100]. Mathematically, the continuous form of the Gaussian function is given by the following [98,99]:

$$G\left(x\right)=\frac{1}{\sqrt{2\pi \sigma^{2}}}e^{\frac{x^{2}}{{2\sigma }^{2}}}$$

where *x* is the position relative to the window center, σ is the standard deviation, which controls the width of the kernel. A practical implementation can be performed using the following [100]:

$$y\left[n\right]=\sum_{i=-k}^{k}x\left[n+i\right]\cdot G\left(i\right)$$

where G is obtained using the abovementioned equation. This approach ensures smooth filtering without introducing modulation artifacts or significant phase distortion [98]. In biomechanical applications, the Gaussian filter has been used to clean signals where both temporal continuity and shape preservation are critical [33].

Madgwick filter

The Madgwick filter is a quaternion-based orientation estimation algorithm commonly used in biomechanical preprocessing to derive reliable angular orientation from IMUs [101,102]. Designed for low-cost microcontroller applications, it fuses data from IMUs to overcome the individual limitations of each sensor, such as drift in gyroscopes or instability in accelerometers during dynamic motion [103]. To this purpose, the Madgwick filter employs a gradient descent optimization approach to minimize the error between the measured and estimated directions of gravity and magnetic field vectors [101]. This results in a real-time estimate of the sensor’s orientation represented as a unit quaternion q. The core update mechanism relies on solving the following differential equation [102]:

$$\dot{q}=\frac{1}{2}q⊗\omega -\beta \nabla F$$

where *q* is the estimated quaternion orientation, ω is the measured angular velocity (gyroscope data), ∇F is the gradient of the objective function representing orientation error based on accelerometer and magnetometer reading, and β is a tuning parameter that controls the convergence rate.

This formulation enables the Madgwick filter to achieve real-time orientation tracking with minimal computational cost. In the context of motion analysis, it is particularly useful during the preprocessing stage to transform raw IMU data into stable orientation signals, which can then be used to further biomechanical analyses, such as joint-angle calculation, gait-event detection, or posture classification [40]. It is especially favored in wearable systems where computational efficiency, low power consumption, and minimal drift are required.

Selection criteria on summary subsection

Table 6 presents a comparative summary of the preprocessing filters and techniques in motion analysis research. The filters are categorized according to their use, such as noise reduction, smoothing, or sensor data fusion.

From the table, some conclusions can be extracted:

- Signal integrity preservation: Some filters, such as SG and Gaussian, are preferred when it is critical to retain the signal’s shape, curvature or peaks (e.g., joint angles and impact events).
- Phase sensitivity: Techniques such zero-lag filters are specifically used when the temporal alignment of events (e.g., foot strike and jump take-off) is required.
- Noise type and distribution: Median filters are effective for sparse, high-magnitude outliers, while Butterworth and moving average filter handle continuous high-frequency noise.
- Real-time vs. postprocessing contexts: Complementary filter, Madgwick filters, and low-order Butterworth implementations are suitable for embedded systems, while computationally heavier methods such as zero-phase filtering are reserved for offline analysis.
- Sensor fusion and orientation estimation: Filters like Madgwick and complementary filters are designed to integrate multiple IMU signals to estimate orientation in real time. These are especially useful in dynamic conditions where both drift correction and computational efficiency are required.

### 3.2. Processing Techniques

After the initial preprocessing stage, where raw motion data are cleaned and standardized, data processing uses a series of computational techniques aimed at enhancing, transforming, or extracting meaningful information from the sensor data. This stage bridges the gap between raw signals and high-level interpretations by enabling the derivation of biomechanically relevant parameters, temporal segmentation, and refined data representation.

Processing techniques may include feature extraction, signal decomposition, state estimation, and sensor fusion, among others. These methods are essential for translating raw measurements, such as position, velocity, or acceleration, into interpretable constructs, like orientation, limb trajectories, joint behavior, or event markers. Moreover, some processing strategies employ model-based approaches, optimization algorithms, or transformation techniques to increase the accuracy and robustness of the results. This section presents the processing strategies identified in this paper, focusing on those that contribute significantly to the preparation of motion data for classification (most commonly) or only validation data.

#### 3.2.1. Kalman Filter

The Kalman filter is a recursive estimation algorithm widely used in motion analysis to predict and correct dynamic states based on noisy sensor measurements and system models [104,105]. Its primary function extends to state estimation and error compensation, particularly when dealing with uncertainty introduced by low-cost or imperfect sensing technologies [5,14,22,23,24,39,57,78].

By design, Kalman filter integrates a prior knowledge of the system dynamics with real-time sensor observations to estimate hidden or indirectly measurable variables such as orientation, joint position, or velocity [105,106]. It operates in two main steps: prediction and correction. During the prediction phase, the filter projects the current state forward in time using a motion model. In the correction phase, it updates that prediction based on new measurements, weighted by their estimated uncertainty. Mathematically, the Kalman filter estimates the system state, ${\hat{x}}_{k}$, and its covariance, $P_{k}$, at time step k using the following recursive equations [105]:

Prediction:

$$\begin{array}{c} {\hat{x}}_{\left.k\right|k-1}={A\hat{x}}_{\left.k-1\right|k-1}+Bu_{k}\\ \;P_{k|k-1}=AP_{k-1|k-1}A^{T}+Q \end{array}$$

Update:

$$\begin{array}{c} K_{k}=P_{k|k-1}H^{T}{\left(HP_{k|k-1}H^{T}+R\right)}^{-1}\\ {\hat{x}}_{\left.k\right|k}={\hat{x}}_{\left.k\right|k-1}+K_{k}\left(z_{k}-H{\hat{x}}_{\left.k\right|k-1}\right)\\ P_{k|k}=\left(I-K_{k}H\right)P_{k|k-1} \end{array}$$

where ${\hat{x}}_{k}$ is estimated state, *A* and *B* are the state transition and control matrices, *Q* and *R* are the processing and measuring noise covariances matrices, $K_{k}$ is the Kalman gain, $z_{k}$ is the measurement at time *k*, and H is the measurement model.

In motion analysis, Kalman filter is particularly valuable for sensor fusion applications, such as combining accelerometer and gyroscope data to estimate orientation, or integration position and velocity data to reduce drift and noise. It is also employed in real- time tracking systems, inverse kinematic, and trajectory reconstruction, where robustness against sensor imperfection is crucial. Despite its advantages, the effectiveness of the Kalman filter depends on system dynamics accurate modeling and noise characteristics. If the assumptions of linearity or Gaussian noise are violated, performance may degrade, in which case extended or unscented Kalman filters can be used as alternatives.

#### 3.2.2. Kabsch Algorithm

The Kabsch algorithm is a mathematical method designed to compute the optimal rigid-body transformation (i.e., rotation and translation) that minimizes the root mean square deviation (RMSD) between two sets of corresponding three-dimensional points [107,108]. In motion analysis, it is particularly useful for aligning motion-capture data, comparing body segment positions, and correcting registration error across different frames or measurements sessions [109]. In [78], the Kabsch algorithm was applied to align sets of 3D markers positions, enabling a more accurate comparison of body shapes or segment orientations.

The algorithm operates under the assumption that there is a one-to-one correspondence between them through an optimal rotation matrix, *R,* computed via singular-value decomposition (SVD).

$$H=\sum_{i=1}^{n}\left(A_{i}-\bar{A}\right){\left(B_{i}-\bar{B}\right)}^{T}\;\left[U,S,\;V\right]=SVD\left(H\right)\;R=VU^{T}$$

where A and B are the point sets, $\bar{A}$ and $\bar{B}$ are the centroids, and H is the cross-covariance matrix. The translation vector *t* is given by the following:

$$t=\bar{B}-R\bar{A}$$

This rotation matrix, *R*, aligns the two points clouds with minimal RMSD [108,109]. If necessary, a translation vector can also be computed to fully superimpose the datasets. The Kabsch algorithm is particularly valuable in biomechanical applications, where shape consistency, joint-position tracking, and motion comparisons are essential, such as gait analysis and postural distortion during alignment, as it supports high-accuracy analysis without introducing scaling or nonlinear warping artifacts [109,110].

#### 3.2.3. The Rauch–Tung–Striebel (RTS) Smoother

The Rauch–Tung–Striebel (RTS) smoother is an extension of the Kalman filter designed to provide more precise and reliable state estimates by incorporating information from both past and future observations [111]. While the Kalman filter performs forward recursive estimation, predicting and correcting the state based only on current and past measurement, the RTS smoother refines these estimates retrospectively, using a backward pass through the data after the forward Kalman filtering process is complete [112].

In [28], the RTS smoother is employed to improve the accuracy of motion data estimations by minimizing uncertainty over entire sequences rather than relying on real-time predictions. This postprocessing refinement leads to lower variance and better noise suppression, especially valuable in offline analyses where full datasets are available. Mathematically, after the Kalman filtering stage generates forward estimates, ${\hat{x}}_{k}$, and error covariances, $P_{k}$, the smother updates them using the following backward recursion [111,112]:

$$\begin{array}{c} G_{k}=P_{k}A^{T}P_{K+1}^{-1}\\ {\hat{x}}_{k}^{smooth}={\hat{x}}_{k}+G_{k}\left({\hat{x}}_{k+1}^{smooth}-A{\hat{x}}_{k}\right)\\ P_{k}^{smooth}=P_{k}+G_{k}\left(P_{k+1}^{smooth}-P_{k+1}\right)G_{k}^{T} \end{array}$$

where $G_{k}$ is the smoother gain, *A* is the state transition matrix, ${\hat{x}}_{k}^{smooth}$ is the smoothed estimate at time k, and $P_{k}^{smooth}$ is the smoothed error covariance.

In the context of motion analysis, RTS is particularly useful for refining the reconstruction of trajectories, estimating joint kinematics, or improving the robustness of multi-sensor fusion results where post hoc processing is feasible [113]. However, due to its requirements for complete data sequences, the RTS smoother is typically limited to offline processing scenarios rather than real-time applications [114].

#### 3.2.4. Processing Techniques Summary

Table 7 presents an overview of the main processing techniques identified in the reviewed literature for motion data analysis. These techniques are selected based on their relevance to key tasks such as state estimation, data alignment, feature extraction, object detection, and data smoothing. The methods are categorized according to their primary application domain, such as state prediction, sensor fusion, or spatial alignment.

Each technique offers advantages and limitations that must be carefully considered when designing motion-analysis strategies:

- Real time vs. offline applicability: Methods such as the Kalman filter enable real-time state estimation, while techniques such as the RTS smoother are restricted to postprocessing scenarios.
- Accuracy and robustness: Methods such as the Kabsch algorithm and Kalman filter provide high accuracy in aligning and estimating states.

### 3.3. Classification Techniques

In motion data analysis, classification techniques play an important role when the goal is to categorize movement patterns, recognize specific motor tasks, detect anomalies, or support clinical decisions based on biomechanical data. They perform these activities by the mapping of processed features or temporal sequences into user-defined classes such as walking phases, rehabilitation states, or performance levels, among others. Their effectiveness depends not only on the selected algorithm but also on the quality of data used for its training.

In general terms, classifications methods are integrated in the final stage of the analysis pipeline. Their objective is to generate discrete or continuous labels that provide actionable insights in biomechanical analysis. From all the assessed articles, the classification strategies can be grouped into two major algorithms:

- Machine learning-based approaches: They rely on statistical models trained on extracted features. Algorithms such as random forest, support vector machines (SVMs), linear regression variants, and discriminant analysis are some examples of the classification approaches that are commonly employed.
- Deep learning-based approaches: They learn the representations directly from raw or minimally processed data. Architectures such as convolutional neural networks (CNNs), long short-term memory networks (LSTMs), and hybrid models are examples of these types of classifiers.

The following subsections describe the classification techniques employed in the revised articles, including their underlying principles, reported applications, and comparative advantages within a biomechanical context.

#### 3.3.1. Deep Learning-Based Classifiers

Convolutional Neural Networks

**Figure 9:**
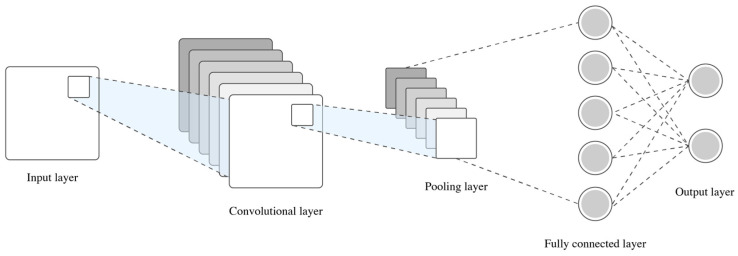
CNN architecture.

Convolutional neural networks (CNNs) are a class of deep-learning architectures particularly effective for analyzing spatially or temporally structured data, such as images or videos [115,116]. In motion analysis, CNNs are applied to automatically learn hierarchical representations from raw or minimally processed data, thus eliminating the need for handcrafted feature extraction. A typical CNN consists of multiple layers, including convolutional layers, pooling layers, and fully connected layers. The convolutional layers apply a set of learnable filters to the input data, producing feature maps that highlight local spatial patterns. Pooling layers reduce the dimensionality of these maps, allowing the network to generalize and focus on the dominant features. Finally, fully connected layers interpret these patterns to produce class predictions or regressions [116]. Figure 9 illustrates the aforementioned topology.

Reference [54] implements a CNN to classify dynamic movement phases using two-dimensional time-frequency representations (i.e., spectrograms), derived from IMU signals. In [35], CNN layers are employed to recognize movement patterns captured by wearables sensors, where convolutional filters extract temporal features associated with gait cycles. Similarly, in [37] CNNs are employed for classifying motor tasks using electromyographic (EMG) signals, allowing spatial filtering of activation patterns. In [117], CNN components are integrated into a more complex U-Net architecture for spatial segmentation, where the time-series maps full-body motion sequences. The U-Net model is a specialized CNN variant featuring and encoder–decoder architecture with skip connections. In the context of biomechanical analysis, both CNN and U-Net models have been proved effective for human movement segmenting phases, classifying motor activities, and identifying transitions in gait or posture [54,117]. Their ability to process high-dimensional sensor data and learn discriminative patterns without manual feature engineering makes them powerful tools in applications such as rehabilitation monitoring, athletic performance analysis, and motor disorder assessment [117].

Long Short-Term Memory Networks (LSTMs)

**Figure 10:**
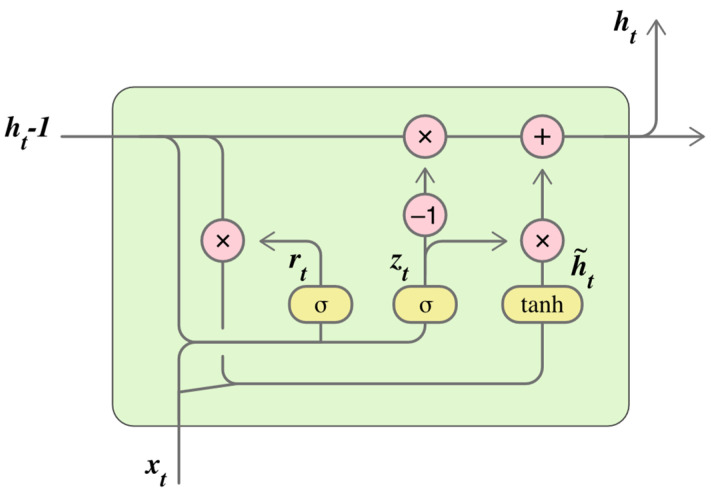
LSTM’s basic architecture. The arrows indicate the information flow through the network.

Long short-term memory (LSTM) networks are a class of recurrent neural networks (RNNs) designed to model sequential data by capturing long-range temporal dependencies [118,119]. Contrary to traditional RNNs, LSTM incorporates internal memory cells and gating mechanisms (input, forget, and output gates) for allowing the network to retain or discard information over time, making them particularly suitable for time-series data such as those generated in motion capture or wearable sensor systems [120]. Figure 10 graphically presents the internal structure of a LSTM cell, which is designed to handle sequential data by controlling the flow of information over time. Figure 10 shows the different components that interact during each time step: the previous hidden state, $h_{t-1}$; the current input, $x_{t}$; and how they are processed through gating mechanisms [118,120].

- The reset gate ($r_{t}$) and update gate ($z_{t}$) regulate how much of the past information should be forgotten or carried forward.
- The tanh block computes a candidate value, ${\tilde{h}}_{t}$, which represents the new information that could be added to the memory.
- A weighted combination between the old state and the new candidate forms the current output, $h_{t}$, allowing the LSTM to retain important information or update it based on new input.

In biomechanical analysis, LSTMs are commonly used to model the temporal evolution of human movement, enabling classification of activities or prediction of motion trajectories based on sequential inputs [8,26,49]. In [8], an LSTM-based regression model is implemented to continuously estimate biomechanical variables over time, demonstrating its effectiveness in capturing smooth transitions within motion patterns. Reference [26] introduces DeepConv-LSTM architecture, which integrates convolutional layers for local feature extraction with LSTM layers for sequence modeling, enhancing the recognition of complex movement patterns in wearable sensor data. Additionally, Reference [49] employed a Bidirectional LSTM (BiLSTM) to process input sequences in both forward and backward directions, allowing the model to leverage full temporal context for prediction. This approach proved particularly useful in applications such as offline gait analysis and rehabilitation monitoring, where understanding both past and future motion trends is critical.

These LSTM-based architectures are especially effective in applications where the sequence and timing of events, such as heel strikes, stance–swing transitions, or joint oscillations, are critical for accurate classification [121]. By learning patterns across entire sequences rather than isolated frames, LSTM models provide a dynamic understanding of movement, making them well-suited for classifying complex motor behaviors and temporal abnormalities [121,122]. In biomechanical contexts, LSTM and its variants have been successfully applied to datasets collected via IMUs, EMG systems, and optical tracking [3,122]. Their flexibility allows for end-to-end learning directly from time-domain signals, supporting biomechanical applications.

#### 3.3.2. Machine Learning (ML)-Based Classifiers

Regression Models

**Figure 11:**
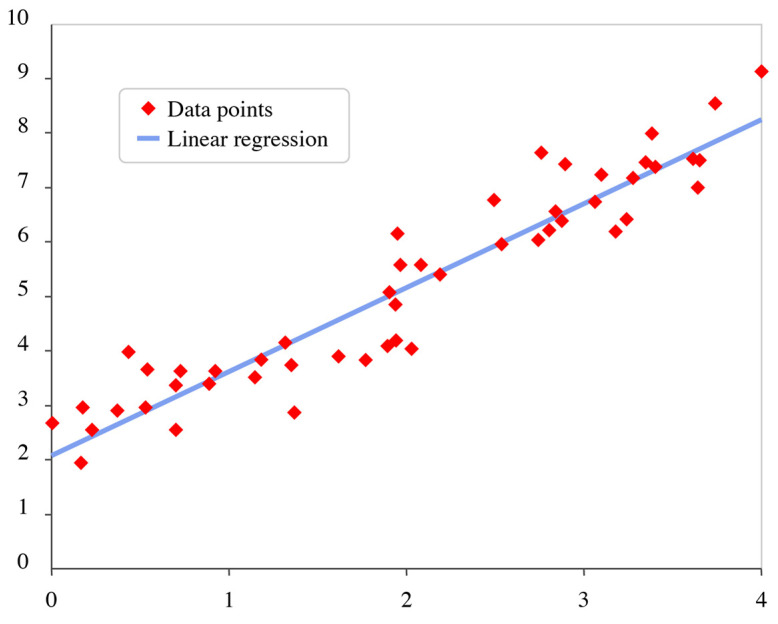
Regression procedure.

In the context of motion data analysis, regression models estimate a continuous or discrete outcome by modeling the relationship between a set of input features and a target variable [123]. Its representation can be seen in Figure 11.

In other words, regression models predict quantitative values or scores, which then can be used directly for the decision-making process [4,15,18,123]. These models are particularly advantageous when the objective is to interpret how changes in biomechanical parameters (e.g., joint angles and force accelerations) influence a specific performance metric or health-related indicator.

For instance, Reference [15] employs linear regression and quantile regression forest models to analyze the variability of biomechanical outcomes across multiple trials, allowing the researchers to estimate distributions rather than single-point predictions, an approach useful for capturing uncertainty in human-movement data. Reference [4] employs a random forest-based regression model to map sensor-derived features onto continuous biomechanical outputs, providing robustness against overfitting and improved handling of nonlinear relationships between variables. In [18], a multilinear relationship model is used to quantify the contribution of individual body segments to global movement variables, enabling insight into the biomechanical coordination of complex activities to be gained.

In biomechanical applications, regression-based models are especially valuable in tasks such as predicting ground reaction forces, estimating joint torques, or modeling center-of-mass trajectories from wearable-sensor data. Their simplicity and interpretability make them suitable for clinical contexts where understanding the contribution of each variable is as important as the accuracy of the prediction. Furthermore, when data are limited or highly structured, regression models offer a lightweight alternative to deep learning, with strong generalization capabilities and reduced computational requirements.

Random Forest Models

**Figure 12:**
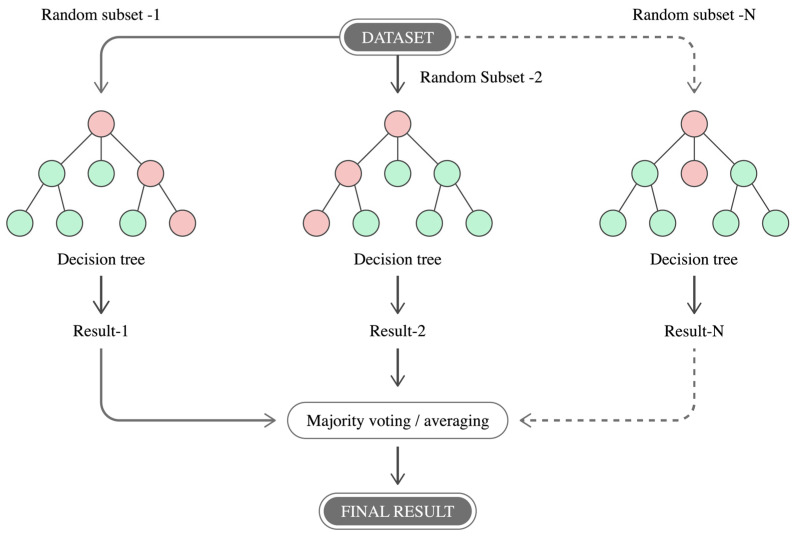
Random forest architecture.

Random forest-based models ensemble-learning techniques that construct multiple decision trees during training (as seen in Figure 12) and aggregate their outputs, either by majority vote (for classification) or averaging (for regression), to produce robust and generalizable predictions [124]. These models are particularly well-suited for biomechanical applications due to their ability to handle high-dimensional input spaces and nonlinear relationships without requiring extensive parameter tuning [124,125].

Reference [4] employs a random forest regression model to estimate biomechanical variables such as joint angles and gait-related features from IMUs. The ensemble approach improved predictive accuracy and mitigated the influence of noise and variability. In [126], a random forest classifier is proposed to categorize different gait phases based on statistical features extracted from inertial data. This model demonstrated high classification accuracy, as it is able to identify subtle differences in motion patterns across subjects and trials.

In the field of motion analysis, random forest models offer several advantages: they are not prone to overfitting when configured properly, can process both continuous and categorical inputs, and provide measure of variable importance, which is valuable for feature interpretation [126]. These features make them particularly effective for tasks such as gait phase detection, activity recognition, and prediction of motion metric in clinical, rehabilitation, or sports performance settings [125,126].

Support Vector Machines (SVMs)

**Figure 13:**
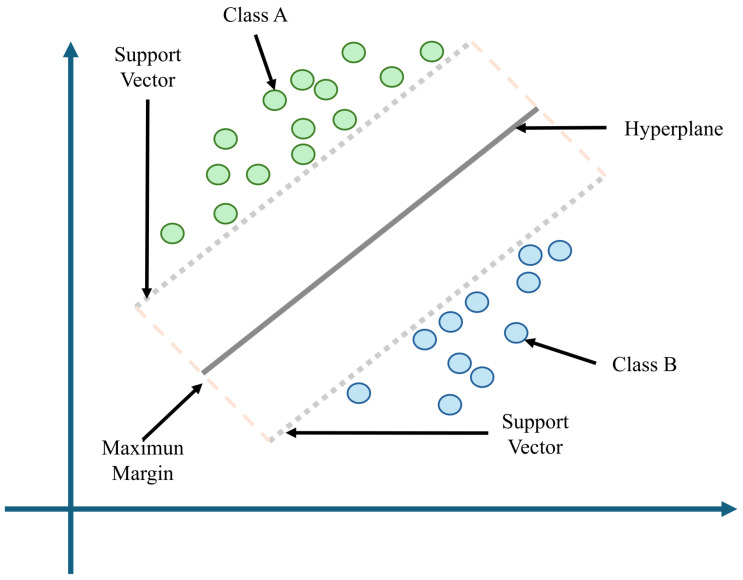
SVM model.

SVMs are supervised models that aim to find the optimal hyperplane that best separates data into classes by maximizing the margin between support vectors, i.e., data points closest to the decision boundary [127]. This feature makes SVMs particularly effective in high-dimensional spaces and in problems where class boundaries are nonlinear, especially when combined with kernel functions [128]. Figure 13 shows the fundamental concept behind a linear SVM, a supervised ML algorithm widely used in biomechanical classification tasks. In this representation, two classes (Class A and Class B) are linearly separable, and the SVM constructs a decision boundary known as the hyperplane (gray line) that best separates the two groups. The optimal hyperplane is defined as the one that maximizes the margin between the two closest data points from each class, referred to as support vectors. This maximum margin enhances the model’s generalization capability when classifying new, unseen data [128].

In [18], SVMs are applied to classify different phases or types of movement based on features derived from multi-sensor motion recordings. The model demonstrated robustness in distinguishing between overlapping motion states, even when feature distributions were not linearly separable. In [5], SVMs are used as classifiers to identify gait patterns and segment movement sequences based on statistical characteristics extracted from the signal. The ability of SVM to generalize from limited data made it a competitive option in this context.

SVMs are frequently used in biomechanical classification tasks where the number of samples is relatively small compared to the number of features, such gait phase detection, activity recognition, or posture classification [5,18,129]. Their effectiveness increases when feature extraction algorithms are employed, allowing developing interpretable classifiers as the decision boundaries can be explained.

Other ML and DL classifiers

In [5], Linear Discriminant Analysis (LDA) is employed to classify gait patterns based on a linear combination of input features that maximizes the separability between classes. LDA is a classical statistical method that projects high-dimensional data onto a lower-dimensional space while preserving class discriminability, making it effective in applications where feature distributions are approximately Gaussian and linearly separable [100]. In [18], Boosted Trees are implemented as an ensemble method to improve classification performance by combining multiple weak learners (typically decision trees). Each subsequent tree focuses on the error made by the previous ones, refining the classification iteratively. This approach is particularly useful in biomechanical analysis due to its capacity to model complex and nonlinear relationships between features and movement classes, while maintaining relatively low computational burden.

In Reference [2], a Feedforward Artificial Neural Network (ANN) is used to classify movement patterns based on extracted features. This architecture, composed of fully connected layers, is capable of learning nonlinear mappings between input features and output labels and is particularly effective when the dataset is well structured and normalized. In [5], the K-Nearest Neighbor (KNN) algorithm is employed for classifying gait patterns by comparing feature vectors with those from labeled instances. Moreover, Reference [73] applies a Nonlinear Autoregressive Network with Exogenous Inputs (NARX) model, which is a recurrent neural network variant designed to capture time-dependent relationships while integrating externals signals, to model and predict biomechanical responses by considering both current and past input–output sequences. These architectures demonstrate that while more complex models like CNNs and LSTMs offer powerful feature abstraction, simpler neural and non-neural classifiers remain relevant, particularly in structured motion-analysis tasks or when interpretability and computational efficiency are priorities. Finally, in Reference [19], a specialized classifier called Feature-Based Variable Environment Statistical Pattern Analysis (F-VESPA) is introduced to adapt classification performance to variable environment conditions. This method integrates statistical pattern recognition with an environmental feature adjustment, making it suitable for wearable systems operating under uncontrolled or real-word conditions. F-VESPA allows for robust classification of human activity patterns by dynamically adjusting to context variability, such as terrain, speed, or sensor displacement.

#### 3.3.3. Classification Techniques Summary

Table 8 presents a comparative summary of the classification models discussed above.

From the table, it is seen that the choice of classifier is influenced by several key criteria, including the following:

- Data complexity and structure: Deep-learning models such as CNN and LSTMSs are the preferred option when input data are high-dimensional or sequential (e.g., time series from IMUs or EMG). In contrast, ML models such as SVMs, random forests, or LDAs are often applied to feature-extracted sets derived from preprocessed or processed data.
- Activity dynamics: Tasks involving subtle motion phases (e.g., gait segmentation or postural transitions) benefit from models that capture temporal dependencies, such as LSTMs or BiLSTMs. For simpler tasks (e.g., identifying activity type), algorithms such as random forest or SVM can perform adequately without degrading the model’s performance.
- Computational efficiency and interoperability: ML models are computational, which, in most scenarios, makes them easier to interpret, while DL models typically require more data and resources but provide superior performance in complex scenarios.
- Accuracy and robustness: Articles that employed DL models generally reported higher classification accuracy, particularly in tasks involving continuous movement predictions, multichannel inputs, or noisy environments.

### 3.4. Algorithms for the Motion Reconstruction and Image Analysis

In modern marker-less motion analysis systems, the trajectories’ reconstruction and velocities from videos rely on pose-estimation algorithms that detect and track anatomical key points over time. These algorithms, typically based on convolutional neural networks (CNNs), identify 2D joint coordinates frame by frame that are then aligned temporally using the video-frame rate. From these time-aligned positions, motion-capture software computes kinematic variables such as trajectory and velocity. Depending on the system configuration, positions may be reconstructed in 2D or triangulated into 3D space using synchronized and calibrated cameras. The methods are implemented in tools such as OpenPose, DeepLabCut, and the Pose2Sim framework [130,131], among the main computer programs.

The core computational principle involves measuring the displacement of each detected joint between consecutive frames. Considering that the frame interval (∆t) is known, software can estimate instantaneous linear velocity as the displacement divided by time:

$$v=\frac{∆x}{∆t}$$

In this sense, the direct extraction of motion variables such as walking speed, limb swing velocity, or step cadence from raw video data can be performed. Software like OpenPose and BlazePose applies this approach to 2D key point trajectories, while systems with multiview capabilities (e.g., AniPose or DeepLabCut with triangulation) extend the method to spatially accurate 3D tracking [132,133].

To enhance robustness, many pose-estimation pipelines incorporate temporal smoothing or biomechanical modeling. For instance, BlazePose-Seq2Seq integrates recurrent neural network structures to refine joint trajectories based on the motion history preceding frames, improving velocity estimation in varying gait conditions [132]. Similarly, DeepLabCut supports transfer learning, where custom-trained networks on specific domain data combined with refinement steps significantly improve the detection and continuity of motion data, particularly for gait assessment [134,135]. These features are essential for minimizing jitter and compensating for transient occlusions or detecting gaps, especially when estimating speed-related variables directly from video.

Validation studies have shown that pose-based systems can estimate temporo-spatial gait variables with high reliability. For example, [130] reported intraclass correlation coefficients above 0.90 for hip and knee velocity estimations using OpenPose, compared with reference marker-based systems. Meanwhile, systems such as Pose2Sim and Theia3D integrate pose estimation with biomechanical moles like OpenSim to produce physically consistent trajectories even under challenging video conditions or environmental constraints [131,136]. This combination of deep learning, computer vision, and biomechanical modeling underpins the growing capability of these tools to translate video content into precise kinematic variables in both research and clinical settings.

### 3.5. Validation Results

For motion analysis, the validation of results is essential for ensuring the credibility, reproducibility, and applicability of the proposed techniques [6,17,34,70,82,85,117]. Given the diversity of acquisition systems, preprocessing and processing strategies, and classification models, it becomes crucial to evaluate performance using quantitative statistical metrics. These metrics allow for objective comparisons across studies and support the development of standardized protocols. Among the most used metrics are Root Mean Square Error (RMSE), Mean Absolute Error (MAE), Normalized RMSE (NRMSE), Mean Absolute Percentage Error (MAPE), coefficient of determination (R^2^), *p*-values, *t*-test, and cross-validation techniques [5,18,35,37,49,73,117,125].

To provide conclusive insights into the presented results derived from the heterogeneous validation approaches, five result-validation categories are set in this work and summarized in Table 9:

- CT (comparison between tests): Evaluates consistency across repeated trials using the same method or device.
- CS (comparison between systems): Compares outputs from different technologies (e.g., IMU vs. MCS).
- CMET (comparison between methodologies): Contrasts analytical or computational techniques applied to the same dataset or system.
- CC (comparison between classes): Validation results across different user groups, conditions, or movement types.
- MP (method proposal).

Studies employing CT [6,17,31,34,54,63,68,70,78,82,84,95,117] tend to emphasize repeatability and protocol standardization, making them candidates for future clinical or industrial deployment. In contrast, studies that focus on CS [3,12,14,22,24,25,27,29,32,34,39,40,41,42,49,51,55,58,67,75,76,81,92,137,138] assess the interoperability of systems, while those that focus on CC [36,38,44,45,47,48,52,60,64,84] focus on performance generalization. Methodological innovation is reflected in MP [20,21,41,46,50,53,61,62,69,72,73], whereas CMET [23,43,71,74,89] explores the impact of technologies and/or techniques.

## 4. Instrumentation, Processing Techniques, and Classification Data Recommendations for Biomechanical Analysis in Different Activities

As is already seen, there is a broad range of technologies, preprocessing techniques, data-processing methods, classification models, and validation categorization. Hence, it is necessary to provide practical recommendations to guide future applications in motion analysis. The appropriate selection of these elements depends on multiple factors, including the type of activity analyzed, the environment conditions (e.g., laboratory, clinical, and field settings), the complexity of motion patterns, the required accuracy and robustness, the availability of computational resources, and the number and quality of data.

This section proposes a structured set of recommendations aimed at supporting the selection of acquisition technologies, preprocessing and strategies, classification approaches, and validation results. Each recommendation is aligned with the technical requirements and challenges commonly encountered in biomechanical applications, providing a practical framework for researchers, engineers and clinicians aiming to optimize motion data-acquisition and -analysis systems.

### 4.1. Selection of Acquisition Technologies

The choice of appropriate acquisition technologies is an important step for ensuring the reliability, precision, and applicability of motion data-analysis systems. From the presented results, it is evident that most current approaches rely on either electrical-based principle (e.g., IMUs and force platforms) or vision-based systems (e.g., optical motion capture and camera-based tracking). Several strategic recommendations can be outlined to guide the selection and optimization of acquisition technologies according to the specific context of application:

- Vision-based systems (e.g., motion capture with reflective markers): The position and configuration of markers are crucial for maximizing tracking accuracy. As demonstrated by recent studies [139,140,141,142], factors such as the markers’ physical dimensions, color contrast relative to environment, and adherence method significantly impact system performance. It is recommended to experiment with different marker designs and layouts during the calibration phase to optimize robustness under the expected operational conditions.
- Marker-less acquisition using AI-based image processing: In scenarios where the environmental constraints limit the use of physical markers, computer vision-based strategies that employ AI offer a viable alternative [143,144,145,146]. These methods often rely on real-time segmentation and detection algorithms to estimate body or joint position across frames. Special attention should be paid to camera specifications, including frame rate, resolution, field of view, and placement, to ensure sufficient image quality for reliable model inference. Recommendations for optimal camera selection and deployment strategies can be found in [147,148,149,150].
- IMUs and force platforms: The implementation of IMUs and force platforms generally requires less infrastructure complexity compared to vision-based systems. However, careful consideration must be given to sensor positioning relative to the body segments or ground reference. For systems based on discrete electronic components, the sensors deliver analog signals, signal amplification, filtering, and conditioning prior to analog-to-digital conversation, and they are essential to preserve signal integrity. Conversely, if digital output is provided, communication protocols such as SPI or I^2^C are commonly employed, and most moder microcontrollers offer native support for these interfaces.
- When designing or selecting novel sensing systems, wireless data-transmission capabilities should be prioritized. Protocols such as Bluetooth, ZigBee, LoRa, or custom RG solutions must be evaluated based on two main factors: the number of data to be transmitted and the required transmission speed. Trade-offs between data rate, energy consumption, and communication range must be carefully balanced according to the deployment environment and application goals.

Overall, acquisition technology selection must be tightly aligned with the technical constraints, environmental conditions, and biomechanical requirements of each project. A careful preliminary evaluation at the system-design stage can significantly enhance data quality and analytical robustness throughout the subsequent processing and classification stages.

### 4.2. Strategy Selection for Preprocessing, Processing and Classification Algorithms

Once data-acquisition technologies have been properly selected, the subsequent stages, namely preprocessing, processing, and classification, play a decisive role in ensuring that the collected motion data can be analyzed with the necessary precision and robustness. Based on the comparative tables presented in Section 3.1, Section 3.2 and Section 3.3, a set of recommendations is proposed. As shown in Table 6, the selection of preprocessing methods depends strongly on the noise and the required fidelity in temporal or spatial features of the acquired data.

- For laboratory environments with controlled conditions (e.g., MCS or force platform recording), Butterworth low-pass filters (4th–10th order) are generally sufficient to mitigate high-frequency noise without introducing significant phase distortion.
- For field applications using wearable IMUs, medial filters or Savitsky–Golay filters are recommended to better handle outliers and preserve critical motion features such as peaks and transitions.
- In real-time systems or embedded applications (e.g., sport performance monitoring), complementary and Madgwick filters are preferable due to their minimal computational burden, a real-time smoothing capability.

The choice of cutoff frequency must be carefully turned according to the movement-frequency content: typically, 6–12 Hz for gait tracking, 10–30 Hz for high dynamic movements (e.g., jumping and running), and lower frequencies (0–5 Hz) for slow or subtle motions (e.g., postural analysis and fine motor control) [8,15,18,19,30,33,45].

On the other hand, for selecting the processing strategies, summarized in Table 7, some recommendations can be performed, depending on the task to be performed.

- In gait analysis and rehabilitation monitoring, Kalman filters and RTS smoother are highly effective for denoising and reconstructing full trajectories when moderate computational resources are available.
- In sports biomechanics, where precise trajectory alignment is critical (e.g., running, kicking, jumping), algorithms such as the Kabsch method enable robust rigid-body alignment of segmented data, even with minor marker displacements.
- When feature extraction is necessary, statistical features such as mean, standard deviation, entropy, and temporal parameters should be prioritized, as demonstrated in [5,125].

As detailed in Table 8, classification model selection depends on the type of biomechanical task and the data structure. In this sense, DL models (i.e., CNN, LSTM, DeepConv-LSTM, and U-Net) should be prioritized when (1) large datasets are available, (2) complex multimodal data are involved (e.g., IMU and EMG), and (3) a fine-grained segmentation is required (e.g., gait phased transitions and postural adjustments). On the contrary, ML models (i.e., random forest, SVM, Boosted Trees, and LDA) are preferable when (1) datasets are limited; (2) features based on representations are already available; and (3) interpretability and fast deployments are important, such as in clinical environments or wearable device applications. The tables synthesized in this review provide a functional guide to navigating these decisions, ensuring that the technological and methodological choices are properly aligned with the intended biomechanical objectives.

### 4.3. Recommendations for the Selection of the Analysis and/or Reconstruction Software

Once the motion data have been acquired, the selection of analysis software becomes a decisive factor in ensuring the reliability and applicability of the reconstructed kinematic information. Based on the findings summarized in Section 3.4, the following recommendations are proposed:

1. Use multiview systems with 3D triangulation in laboratory settings requiring high spatial accuracy. For controlled environments equipped with multiple calibrated cameras, software such as DeepLabCut (with triangulation) or Pose2Sim are recommended. These systems offer accurate 3D reconstruction by merging multiple 2D detections and are compatible with biomechanical modeling environments like OpenSim, allowing for extended analyses such as inverse dynamics or joint moment estimation [131,135].
2. Select monocular, lightweight tools for field or clinical scenarios with minimal hardware infrastructure. In scenarios where only a single RGB camera is available, or where user accessibility is prioritized (e.g., rehabilitation clinics and sports fields), pose estimation framework like OpenPose, BlazePose, or DeepLabCut with pretrained models are suitable options. Among these, BlazePose has demonstrated efficiency for mobile or real-time deployment scenarios due to its low computational burden [132,133,136].
3. Systems with internal filtering and refinement for accurate estimation should be selected. When the biomechanical objective includes computing velocity or temporo-spatial parameters (e.g., step time and swing speed), it is essential to use software that stabilizes key point trajectories. In this sense, systems that integrate Kalman filters, spline-smoothing strategies, or RNN-based refinement models, as such BlazePose-Seq2Seq or custom-trained DeepLabCut, improve the robustness of motion reconstruction, especially during fast or irregular movements [132,134,135].

These three strategies can serve as a practical guide to align the software selection process with the specific biomechanical specifications, available resources, and expected outcomes of a given motion analysis application.

### 4.4. Validation Strategy Selection

The selection of a validation strategy must be closely aligned with the type of study, intended application, complexity of the systems, and availability of ground-truth or reference data. Based on the data described in Table 9, the following guidelines are proposed:

1. The CT strategy can be proposed when the objective is to assess the repeatability and robustness of system or method under the same operational conditions.
2. CS strategies are appropriate when introducing new devices or alternative technologies and benchmarking them against established gold standards.
3. When the focus is on evaluating different data-processing pipelines or analytical techniques on the same datasets, CMET strategies are preferable. Examples include the testing of different feature extraction methods, or the comparison of classification models.
4. When the goal is to differentiate between groups, CC strategies emphasize generalization capability, ensuring that the model or system is not overly tailored to a specific subset of data.
5. MP validations are required when introducing new acquisition systems, preprocessing techniques, processing algorithms, or classification models. Since these studies propose innovations, the validation must rigorously demonstrate the following: (1) performance under different conditions and (2) comparison against baseline methods.

Ensuring rigorous and context-appropriate validation is essential to guarantee that motion analysis results are not only statistically significant but also practically meaningful and applicable across diverse environments.

## 5. Final Remarks and Conclusions

This review was developed with the aim of providing a comprehensive and structured framework for the selection and application of technologies, data-processing strategies, classification models, and validation methods in motion data analysis. By systematically evaluating existing approaches, we can bridge the gap between technological diversity and practical implementation requirements in various biomechanical contexts.

A central contribution of this work is not only the revision of technologies but also the integration of recommendations across acquisition, preprocessing, processing, classification, and validation in relation to the environment (clinical, sport science, and field) and movement type (gait, rehabilitation tasks, and dynamic sports actions). This perspective enables researchers, clinicians, and engineers to design or select motion analysis systems that are technically robust, application-focused, and adapted to real-world constraints.

Across the reviewed studies, DL models, notably CNNs; LSTM variant; and hybrid architectures such as DeepConv-LSTM demonstrated the highest classification and regression accuracy, especially in scenarios involving (1) complex, continuous motion (e.g., gait segmentation and joint angle estimation); (2) multimodal inputs, such as combined IMU and EMG signals or visual time-series data; and (3) real-world or noisy conditions, where handcrafted features alone may fall short.

On the other hand, ML models like random forest, SVM, and LDA provided competitive results in structured and well-defined classification tasks, particularly when (1) feature extraction was already performed during the preprocessing or processing stage and when (2) real-time computation and ease of deployment were important.

While DL models often outperform traditional methods in accuracy, they require more computational resources, larger labeled datasets, and careful tuning. In contrast, ML models offer faster training, simpler implementation, and, often, more transparent decision-making logic.

In conclusion, this article offers a technical roadmap for the structured development and application of motion analysis solutions, supporting informed decision making at every stage of the development process. As the field continues to evolve, particularly with the increasing integration of AI and portable sensing technologies, the need for clear, practical, and technically grounded frameworks will become even more critical for advancing both research and real-world biomechanical applications.

## Figures and Tables

**Table 1:** Overview of motion-capture systems.

System	Type	Recommended Environment
VICON (Vicon T40, Vero, Vantage V5, Nexus V2, MX-F40)	Optical, with markers.	Laboratory, clinical analysis.
Qualisys (Oqus 7+, Oqus 4-series, QualisysTrack Manager)	Optical, with markers.	Laboratory.
Motion Analysis (Hawk, Raptor-4, Smart-Dx)	Optical with marker.	Laboratory, clinical analysis.
Microsoft Kinect V1/V2	Depth sensor.	Field, practical applications.
Azure Kinect	Depth sensor.	Field, practical applications.
Orbbec Astra	Depth sensor.	Field, education.
Smart-Dx (BTS Bioengineering, Milan, Italy)	Photogrammetry.	Laboratory.
GoPro Hero 3, standard webcams	Conventional cameras.	Field, prototypes, low-cost use.

**Table 2:** Overview of commercial inertial measurement units (IMUs).

System	Type	Recommended Environment
Xsens^®^ (MTw, Awinda, MTx, MVN, and DOT)	Commercial IMU system.	Laboratory, field, clinical analysis.
GaitUp Physilog^®^ 5	Wearable IMU system.	Clinical analysis.
RehaGait^®^ Hasomed	Medical IMU platform.	Clinical analysis, rehabilitation.
Notch^®^ IMU system	Wearable IMU system.	Research, sports science.
MyoMOTION Research PRO and Clinical	Research grade IMU system.	Research, sports science.
IMeasuredU	Sport focused IMU system.	Field, sport science.
x-IMU (x-io Technologies, Bristol, UK)	Developer grade IMU.	Research, prototyping.
BTS G-WALK	Portable gait system.	Clinical analysis.
Portabiles GmbH (custom IMUs)	Research and prototypes/high-end IMU.	Research, prototyping.
LORD MicroStrain (3DM-CX5-25, GX4-25)	Research and prototypes/high-end IMU.	Research, prototyping.

**Table 3:** Overview of commercial force platforms.

System	Type	Recommended Environment
Kistler 92811CA	Piezoelectric.	Laboratory, clinical analysis.
Bertec FP6012fi	Strain gauge-based.	Laboratory
Bertec treadmill	Strain gauge-based.	
AMTI BP400600	Strain gauge-based.	
BTS P6000	Optoelectronic.	Laboratory, clinical analysis.

**Table 4:** Alternative sensing technologies.

System	Type	Recommend Environment
CyberGlove^®^ (CyberGlove Systems LLC)	Flex sensor glove.	Laboratory, fine motor control.
BioStampRC (MC10 Inc. (Cambridge, MA, USA))	Wearable stretch sensor.	Daily activities, sport sciences.
Pedar^®^ system (Novel GmbH (Munich, Germany))	Pressure insole.	Laboratory.
Capacitive stretch sensors (Parker Hannifin (Cleveland, OH, USA))	Stretch sensor.	Laboratory, sport sciences.
STR C-STRETCH^®^	Pressure sensor.	Laboratory.
FlexiForce	Stretch sensor.	

**Table 5:** Acquisition technologies’ advantages and disadvantages.

Technology	Activity	Advantages	Disadvantages
MCS	Walking, cuttings, running, football, swimming, squads, martial arts, hand movement, upper and lower limbs, baseball.	High spatial and temporal resolution. Enables full-body 3D biomechanical analysis. Gold standard in controlled environments.	High cost (hardware and software). Controlled operational conditions. Marker displacement
IMU	Walking, cuttings, running, football, swimming, neck, upper and lower limbs, martial arts.	Reduced size. Usable in both indoor and outdoor environments. Real-time 3D orientation data.	Prone to drift and bias accumulation. Requires frequent calibration. Spatial positioning capabilities
FP	Walking, cuttings, running, football, swimming, martial arts.	Direct measurement of forces and torques. High precision for impact and contact analysis. Useful for inverse dynamics calculations.	Fixed setup, Measures forces only at the surface. High initial investment and installation constraints
Other prototypes	Walking, martial arts, running, baseball, squads, hand movements, upper and lower limbs.	Adaptable to irregular surfaces. Enable novel sensing modalities.	Require development, calibration and validation. May lack standardization and durability Limited to experimental proofs

**Table 6:** Overview of preprocessing techniques used in motion data analysis reviewed.

Method	Application	Advantages	Disadvantages
Butterworth low-pass filter	Attenuates high-frequency noise in time-series signals	Smooth frequency response. Easiness of implementation.	Introduces phase delay
Median filter	Removes spikes and outliers, especially in kinetic data.	Preserve edges and discontinuities. Effective for salt and pepper noise.	Not ideal for signals with high variance or slow-changing trends.
MA filter	Smooth data fluctuations to highlight.	Simple and fast implementation. Useful for trend estimation.	Can blur sharp transitions.
Savitzky–Golay filter	Smooths data while preserving local features such as peaks or slopes.	Maintains curvature and sharp features.	Complex parameter tuning. Performance is sensitive to window size
Zero-lag low-pass filter	Smooths data without affecting phase characteristics.	Maintains temporal accuracy.	Requires forward-backward processing. Not suitable for real time applications
Gaussian filter	Reduces high-frequency noise using Gaussian-weighted averaging.	Preserves overall signal shape and continuity.	May blur rapid transitions if the window is too wide.
Complementary filter	Fuses data from sensor to estimate orientation.	Compensation for drift and noise. Suitable for embedded real time systems	Requires calibration Sensitive to rapid transitions on motion dynamics.
Madgwick filter	Sensor fusion algorithm for real time orientation estimation using IMU data.	Provides accurate and drift resistant orientation in harsh conditions. Low computational cost.	Sensitive to initial calibration. Performance may degrade with sensor noise.

**Table 7:** Overview of processing techniques used in motion data analysis reviewed.

Method	Application	Advantages	Disadvantages
Kalman filter	Estimates and predicts signal states by combining measurements with a model.	Provides real-time estimation, smoothing and sensor fusion capabilities.	Requires careful model tuning. Can be computationally intensive.
Kabsch algorithm	Aligns two sets of 3D points by minimizing RMSD.	Highly accurate for rigid-body alignment. Preserves spatial relationships.	Computationally intensive for large point sets. Sensitive to outliers.
Rauch–Tung–Striebel (RTS) smoother	Refines Kalman filter estimates by using future measurements for backward smoothing.	Reduces estimation error. Improves consistency over full motion sequences.	High memory and computational requirements. Only applicable to offline processing.

**Table 8:** Overview of classification techniques used in motion data analysis reviewed.

Method	Application	Advantages	Disadvantages	Results
Artificial neural network (ANN)	Prediction of ground reaction force (GRFs) and range of motion to evaluate gait [2].	Captures nonlinear relationships. Simple architecture.	Requires large datasets. Limited temporal modeling	P = 0.96 ± 0.03 (GRF) P = 0.99 ± 0.03 (°)
Quantile regression forest (QRF).	Prediction of ground reaction force (GRF), vertical impulse, and ground contact time in runners at different speeds [15].	Estimates the uncertainty for each prediction. Robust to outliers.	Computationally intensive with large datasets.	RMSE = 0.150
Linear regression (LR)	Prediction of ground reaction force (GRF), vertical impulse, and ground contact time in runners at different speeds [15].	Easy to implement and interpret.	Limited to linear relationships. May underfit.	RMSE = 0.139
Random forest-based regression model	Estimation of ankle joint power using data from two IMUs on the foot and shank [4].	Handles nonlinearities. Robust to noise.	Reduce interpretability.	R^2^ = 0.94 RMSE = 0.03 NRMSE = 0.49%
Long short-term memory (LSTM)-based regression model	Estimation of ground reaction force (GRF) during stair ascent and descent using kinematic data [8].	Effectively captures both past and future temporal dependencies in sequential motion data.	High training requirements. Unsuitable for small datasets.	RMSE = 3.29%
Random forest	Estimation of muscle levels in runners [126].	Effective on heterogeneous data. Good generalization.	Lack of explainability of the resulting model.	RMSE = 0.06
Multilinear regressor (MLR)	Prediction of turning direction, speeds, and mechanical work in cuttings maneuvers [18].	Computationally efficient. Interpretable coefficients.	Assumes linear relationships between variables. Low performance on complex patterns.	R^2^ = 0.53
Support vector machine (SVM)	Prediction of turning direction, speeds, and mechanical work in cutting maneuvers [18].	Works well with high-dimensional features. Effective in capturing complex relationships.	Sensitive to parameter selection. Fine-tuning selection required.	R^2^ = 0.65
Boosted Trees (BTs)	Prediction of turning direction, speeds, and mechanical work in cuttings maneuvers [18].	Strong performance. Handles noise better than single trees.	Prone to overfitting, especially with noisy data.	R^2^ = 0.6
Convolutional neural network (CNN)	Detect variations and different conditions in the walk [19].	Effective in capturing spatial and temporal patterns in data. Good generalization on image-like inputs.	Require large amounts of labeled data for training. Computationally intensive.	Acc = 90%
	Estimation of joint angles in the sagittal, frontal, and transversal planes during running [35].			R2 = 0.97 RMSE = 2.2° NRMSE = 4.57%
	Detection of anomalous kick in taekwondo competitions [37].			Acc = 95.83%
K-Nearest Neighbors (KNNs)	Prediction of the most relevant factors in chronic neck pain (CNP) [5].	Can adapt to various types of data and patterns. Flexible and intuitive.	Prediction is slow in large datasets. Sensitive to data scaling and noise.	Acc = 84.22%
Support vector machine (SVM)		High accuracy. Can capture complex decisions boundaries.	A fine-tuning is required. Costly for large datasets.	Acc = 86.85%
Line Discriminant Analysis (LDA)		Reduces dimensionality while preserving class separation.	Assumes Gaussian distribution and equal covariances.	Acc = 81.6%
Foot vertical and sagittal position algorithm (F-VESPA)	Detection of foot-strike in gait [19].	Adapts to real-word and real-time conditions.	Specific designed to detect foot strike events. Nor easily generalizable	MAE(SD) = 4.36 (0.41)
DeepConv-LSTM	Model for predicting join angles during movements using IMUs [26].	Integrates spatial–temporal learning. Accurate on sequences.	Demands long training time and annotated data. Required large volumes of datasets for training.	R = 0.67~0.99 MAE = 2.2°~5.1°
BiLSTM	Knee injury detection to people with osteoporosis [49].	Captures bidirectional temporal dependencies.	Requires long sequences and extensive computational.	RMSE = 7.04°~11.78° MAE = 5.99°~10.37° R = 0.85~0.99
Nonlinear autoregressive with exogenous input (NARX) neuronal network	Prediction of dynamic systems and time-series data [73].	Uses historical and exogenous data. Model nonlinear systems.	Complex to optimize. Requires careful tuning of parameters.	RMSE = 4.5°~2.5°
U-Net	Detect specific events on the snowboard [116].	Captures multiscale patterns with spatial precision.	High computational-memory demand. Training needs extensive and labeled data.	Mean Hausdorff = 80.34%

**Table 9:** Result-validation categories for biomechanical analysis used in the papers reviewed.

Method	Uses	Advantage	Disadvantage
CT	Validates consistency across multiple trials using the same setup or protocol.	Demonstrates intra-system repeatability and robustness.	Limited to controlled conditions. External benchmarking is missing.
CS	Compares output from different motion acquisition technologies or hardware platforms.	Support interoperability; evaluates cross-device performance.	May introduce bias due to differences in device specs or calibration.
CMET	Evaluates impact of different algorithms or processing pipelines applied to the same data.	Highlights methodological sensitivity and helps identify optimal strategies.	Requires precise alignment and comparable configurations.
CC	Validates system performance across multiple categories (e.g., user groups and motions).	Reveals generalizability and class-specific performance.	Demands larger and well-balanced datasets for statistical power.
MP	Introduces and validates a novel analytical, processing, or classification approach.	Enables innovation and progress in motion analysis methodology.	New methods may miss baseline comparison or long-term validation frameworks.
